# Omega-Class Glutathione Transferases of Carcinogenic Liver Fluke, *Clonorchis sinensis*, Modulate Apoptosis and Differentiation of Host Cholangiocytes

**DOI:** 10.3390/antiox10071017

**Published:** 2021-06-24

**Authors:** Chun-Seob Ahn, Jeong-Geun Kim, Insug Kang, Yoon Kong

**Affiliations:** 1 Department of Molecular Parasitology, Samsung Medical Center, School of Medicine, Sungkyunkwan University, Suwon 16419, Korea; feg123@skku.edu (C.-S.A.); jgkim82@skku.edu (J.-G.K.); 2 Department of Biochemistry and Molecular Biology, School of Medicine, Kyung Hee University, Seoul 02447, Korea; iskang@khu.ac.kr

**Keywords:** *Clonorchis sinensis*, omega-class glutathione transferase, cholangiocyte, oxidative stress, programmed cell death, cell differentiation, protein kinase, gene regulation

## Abstract

The small liver fluke *Clonorchis sinensis* causes hepatobiliary ductal infections in humans. Clonorchiasis is characterized histopathologically by ductal dysplasia, hyperplasia and metaplasia, which closely resembles cholangiocarcinoma (CCA). The disruption of programmed cell death is critical for malignant transformation, while molecular events underlying these phenomena have poorly been understood in clonorchiasis-related CCA tumorigenesis. We incorporated recombinant *C. sinensis* omega-class glutathione transferase (rCsGSTo) 1 or 2 into human intrahepatic biliary epithelial cells (HIBECs) and analyzed pathophysiological alterations of HIBECs upon the application of oxidative stress. rCsGSTos partially but significantly rescued HIBECs from cell death by inhibiting oxidative stress-induced apoptosis (*p* < 0.01). rCsGSTos modulated transcriptional levels of numerous genes. We analyzed 13 genes involved in programmed cell death (the upregulation of five antiapoptotic and two apoptotic genes, and the downregulation of one antiapoptotic and five apoptotic genes) and 11 genes associated with cell differentiation (the increase in seven and decrease in four genes) that showed significant modifications (*p* < 0.05). The induction profiles of the mRNA and proteins of these differentially regulated genes correlated well with each other, and mostly favored apoptotic suppression and/or cell differentiation. We detected increased active, phosphorylated forms of Src, PI3K/Akt, NF-κB p65, MKK3/6 and p38 MAPK, but not JNK and ERK1/2. CsGSTos were localized in the *C. sinensis*-infected rat cholangiocytes, where cytokeratin 19 was distributed. Our results demonstrated that CsGSTos excreted to the biliary lumen are internalized and accumulated in the host cholangiocytes. When cholangiocytes underwent oxidative stressful condition, CsGSTos appeared to be critically involved in both antiapoptotic process and the differentiation of host cholangiocytes through the regulation of target genes following the activation of responsible signal molecules.

## 1. Introduction

Clonorchiasis, caused by the small liver fluke *Clonorchis sinensis*, represents one of the major fish-borne zoonotic helminthiases. Humans are infected by eating improperly cooked freshwater fish infected with the metacercariae. The disease is prevalent in several Asian countries, where local ethnic dishes made of raw/undercooked freshwater fish are popular and widely consumed. About 15 million people are infected and another 600 million people are at risk annually [1].

Mild infections with *C. sinensis* are usually asymptomatic, but chronic cumulative infections can invoke serious illnesses, such as recurrent pyogenic cholangitis, jaundice, cholelithiasis and cholecystitis. More importantly, the histopathological examination of these patients frequently shows inflammation-associated dysplasia, adenomatous hyperplasia and mucin-secreting metaplasia of the biliary ductal epithelium, and ductal dilatation combined with periductal fibrosis, which closely resembles cholangiocarcinoma (CCA) [2]. Epidemiological evidence indicates that CCA incidence is significantly high in areas where clonorchiasis is endemic, and approximately 10% of CCA is associated with chronic clonorchiasis [3]. CCA may have numerous different predisposing factors; however, common characteristics among these risk factors include chronic inflammation of the biliary epithelium and bile stasis [4]. In several Asian enclaves, chronic cumulative infections with small liver flukes, such as *C. sinensis* and *Opisthorchis viverrini*, are the leading causes of CCA [5]. In some European and Siberian communities, *O. felineus* infection constitutes a major risk factor for CCA development [6,7]. Together with environmental stressors, the mechanical damage of the infecting worms and immunopathological and biochemical cellular disturbance caused by the parasite’s secretions trigger biliary ductal injuries, which may lead to malignant transformation [8,9]. *C. sinensis* and *O. viverrini* are group 1 biocarcinogens [10].

To cope with hostile intraluminal micromilieu of the host bile duct, *C. sinensis* is equipped well with a diverse repertoire of antioxidant enzymes, including glutathione transferases (GSTs). In the *C. sinensis* genome, 12 distinct isozymes of GSTs that belong to six different classes exist. *C. sinensis* has been shown to differentially regulate the secretion of different GSTs in response to bile and endogenous/exogenous oxidative stressors, which suggested that different GSTs might have evolved differentially, each with specialized functions to combat harmful environmental conditions [11,12,13].

Omega-class GST (GSTo) is a cytosolic enzyme that shows distinct structural and functional properties. GSTo is preserved well across wide phylogenetic taxa, but it has only recently been characterized. In addition to antioxidant properties, the physiological functions of GSTo include multidrug resistance, the activation of interleukin-1β, the regulation of signal molecules involved in c-Jun N-terminal kinase (JNK)-mediated apoptosis and the modulation of mitogen-activated protein kinase (MAPK) in response to H_2_O_2_-induced toxicity. It also mediates the sequestration of byproducts generated through hepatic metabolisms [14,15,16].

*Clonorchis sinensis* has two GSTo isoforms: CsGSTo1 and 2. These enzymes are specifically expressed in the reproductive system and play important roles in the protection of these organs during maturation and the response to oxidative stress [13]. Previous studies demonstrated that murine intrahepatic biliary epithelial cells cultured in the presence of *C. sinensis* excretory-secretory products (ESP) promoted the secretion of tumor necrosis factor-α through activation of Toll-like receptor 4 [17]. Human CCA cell lines (HuCCT1) cocultured with cholangiocytes (H69) increased migration and invasion upon treatment with *C. sinensis* ESP [18]. The stimulation of HuCCT1 cells with *C. sinensis* ESP induced the differential secretion of pro-/anti-inflammatory cytokines [19]. These collective data indicated that biologically active *C. sinensis* ESP can exert effects on regional host cells during host–parasite interactions. CsGSTos are secreted into the host environment and elicit specific antibody responses in infected individuals [11,20], while other biological functions relevant to these enzymes in the host remain to be understood.

We hypothesized that excretory CsGSTos could be taken up by host cholangiocytes, which constitute the first-line effector system that interacts with *C. sinensis*. We observed that excretory CsGSTos were accumulated in host biliary ductal cells during the course of infection. We analyzed the biological consequences of CsGSTos within the host cholangiocyte by the in vitro uptake of recombinant CsGSTos (rCsGSTos) into human intrahepatic biliary epithelial cells (HIBECs). Our results demonstrated that rCsGSTos increased the active phosphorylated forms of Src, PI3K/Akt and NF-κB p65 signal molecules, as well as those of MKK3/6 and p38 MAPK, thus modulating the transcriptional and translational expression of a diverse set of genes involved in programmed cell death and cell differentiation.

## 2. Materials and Methods

### 2.1. Rat Biliary Ductal Epithelium Experimentally Infected with C. sinensis

Each of 100 *C. sinensis* metacercariae, which had been collected from naturally infected freshwater fish, were infected with 4- to 6-week-old Sprague Dawley rats. The rats were serially sacrificed from 1 to 24 weeks post-infection. Infection patency was confirmed by worm recovery. The biliary ductal epithelium was collected, homogenized in lysis buffer (Pro-Pre, iNtRON, Seongnam, Korea), centrifuged at 20,000× *g* for 1 h at 4 °C and stored at −80 °C. All protocols for animal experiments were approved by the Institutional Review Board of Sungkyunkwan University (protocol no. 2016-16-4). All animals were housed in AAALAC approved animal facilities in accordance with relevant guidelines and regulations.

### 2.2. Expression of rCsGSTos

The nucleotide sequences of CsGSTo1 (ANK78262) and 2 (ANK78263) were amplified from an adult *C. sinensis* cDNA library. The sequences were verified by sequencing, cloned into pET-28a(+) vector (Novagen, Madison, WI, USA) and transformed into *Escherichia coli* BL21 (DE3) (Thermo Fisher Scientific, Waltham, MA, USA). Bacterial cells were cultured with Luria–Bertani medium supplemented with kanamycin (50 µg/mL). Recombinant proteins, induced with 0.1 mM isopropyl-β-D-1-thiogalactopyranoside, were purified by a nickel–nitrilotriacetic acid column (Qiagen, Hilden, Germany), followed by thrombin cleavage. The proteins were delipidated using an Octyl-Sepharose 4 Fast Flow column (GE Healthcare, Little Chalfont, UK) and bacterial endotoxin was removed using the Endotoxin Removal System (GenScript, Piscataway, NJ, USA). The homogeneity of the recombinant proteins was monitored by 15% reducing SDS-PAGE. Enzyme activity was assayed using dehydroascorbate substrate [13].

### 2.3. Antisera

Polyclonal monospecific antibodies against rCsGSTo1 and 2 were generated in 6-week-old female BALB/c mice. The recombinant proteins (100 μg) mixed with 2% aluminum hydroxide gel adjuvant (Thermo Fisher Scientific) were subcutaneously immunized 3 times with 2-week intervals. One week later, a final booster was administered through intravenous route (10 μg). Blood was collected by heart puncture. IgG fractions were purified by a Protein G affinity column.

### 2.4. HIBEC Culture and Uptake of rCsGSTos

HIBECs (1 × 10^6^ cells; ZenBio, Durham, NC, USA) were seeded on plates and grown in HIBEC growth medium (IHBEC-1, ZenBio) for 48 h at 37 °C under 5% CO_2_ atmosphere [21]. Culture medium was exchanged with serum-free RPMI-1640 and cells were serum-fasted for 2 h at 37 °C. The cells were then incubated with serum-free RPMI-1640 supplemented with either rCsGSTo1 or 2 (10, 25 or 50 μg/mL) for 1 h, after which, the culture medium was changed to IHBEC-1. The absorption of rCsGSTos by HIBECs was determined by immunocytochemical staining. A portion of the HIBECs incubated with respective rCsGSTo was transferred onto a fibronectin-coated glass slide and stabilized for 30 min. Cells were fixed in 2% paraformaldehyde for 10 min and permeabilized with 0.5% Triton X-100 for 5 min. Cells were incubated with anti-rCsGSTo1 or 2 antibodies (1:250 dilution) overnight at 4 °C and additionally with 1:1000 diluted FITC-conjugated goat anti-mouse IgG antibodies (Abcam, Cambridge, UK) for 2 h. Nuclear staining was carried out with propidium iodine (PI) or 4′,6-diamidino-2-phenylindole (DAPI) (Abcam). Images were obtained under an IX71 inverted microscope (Olympus, Tokyo, Japan).

### 2.5. Oxidative Stress

HIBECs (1 × 10^6^ cells) that were incorporated with 50 µg/mL of rCsGSTo1 or 2 were transferred to fresh IHBEC-1 medium and stabilized for 1 h. The cells were exposed to different doses of cumene hydroperoxide (CHP; Sigma-Aldrich, St. Louis, MO, USA) from 50 to 300 μM and/or for different time intervals from 30 min to 72 h at 37 °C in a 5% CO_2_ incubator. Cells cultured at each condition were harvested and used in the following experiments. All experiments involving oxidative stress were independently performed at least 3 times with freshly prepared different batches of cells.

### 2.6. Quantitative RNA Sequencing (Quant-RNAseq)

Total RNA isolated from each of the conditioned HIBECs was qualified with an ND-2000 spectrophotometer (Thermo Fisher Scientific) and Agilent 2100 bioanalyzer with the RNA 6000 Nano Chip (Agilent Technologies, Santa Clara, CA, USA). cDNA libraries were constructed using the QuantSeq 3′ mRNASeq Library Prep Kit (Lexogen, Vienna, Austria). Total RNA (500 ng) was hybridized with oligo dT primer containing an Illumina compatible sequence on the 5′-end and reverse-transcribed into cDNA. The RNA template was degraded and cDNA synthesis was initiated by a random primer containing an Illumina compatible linker sequence. The cDNA library was purified using magnetic beads and amplified with the complete adapter sequences required for cluster generation. The amplified library was purified from PCR components. We selected library fragments longer than 200 bp in size. Their sizes and qualities were verified by a Bioanalyzer 2100 (Agilent Technologies). High throughput sequencing was performed as a single-end 75 sequencing by NextSeq 500 (Illumina, San Diego, CA, USA).

### 2.7. Quantitative Real Time Reverse Transcription-PCR (qRT-PCR)

Total RNA (200 ng) mixed with gene-specific primers (Table S1) was applied for qRT-PCR with a QuantiTect SYBR Green RT-PCR Kit (Qiagen). qRT-PCR conditions were as follows: reverse transcription for 30 min at 45 °C, PCR initial activation for 15 min at 95 °C, followed by 45 PCR cycles of 15 s at 94 °C, 30 s at 50 °C and 30 s at 72 °C with melting curve analysis. The β-actin gene was used as the control. The relative expression was normalized with the control gene and calculated by differences in cycle threshold (Ct) using Rotor-Gene ScreenClust HRM Software (Qiagen).

### 2.8. Data Analysis

Reads of QuantSeq 3′ mRNASeq were aligned through Bowtie2 (http://bowtie-bio.sourceforge.net/, accessed on 18 April 2019). Bowtie2 indices were generated from the representative transcripts for aligning to the transcriptome. These files were used to assemble transcripts by estimating their abundances and to detect differential transcriptome expressions. Differentially expressed genes were determined by counts of unique and multiple alignments with coverage in Bedtools (https://bedtools.readthedocs.io/, accessed on 18 April 2019). The read count (RC) data were processed by quantile normalization using Bioconductor inserting EdgeR within RC. Transcripts were identified by a Medline database search (http://www.ncbi.nlm.nih.gov/, accessed on 18 April 2019). To ensure confidence in our selection and reproducibility and to compare expressional changes between the control and stressed cells, we applied data extraction factors with fold change ≥ 2.00 and normalized RC data [log2] ≥ 4.00 (*p* < 0.05). Expression profiling of transcripts was visualized by MultiExperiment Viewer (MeV) analysis (http://mev.tm4.org/, accessed on 26 March 2020). A gene tree was created with log2 Transform of Adjust Data, Euclidean Distance Metrics and Hierarchical Clustering. Transcriptional diversity was categorized by double gradient from low (−5.0) to upper limits (5.0). Gene ontology and Kyoto Encyclopedia of Genes and Genomes (KEGG) pathways were analyzed by Blast2GO (https://www.blast2go.com/, accessed on 27 March 2020) and KEGG mapper (https://www.genome.jp/kegg/mapper.html, accessed on 1 April 2020). Gene set enrichment analysis (GSEA) was performed on 412 genes extracted from rCsGSTos-incorporated HIBECs under oxidative stressful conditions, employing a gene sets database of c5.all.v7.2.symbols and a chip platform of Human_Gene_Symbol_with_Remapping_MSigDB.v7.2 (https://www.gsea-msigdb.org/gsea/index.jsp, accessed on 3 December 2020). To analyze the statistical significance of GSEA between groups, 100 permutation was assigned to calculate the enrichment score (ES), normalized ES (NES), nominal *p*-value, a false-discovery rate (FDR) *q*-value and a family-wise error rate (FWER) *p*-value on weighted_p2 with Signal2Noise.

### 2.9. Double Sandwich-ELISA

Wells of microtiter plates (Greiner Bio-One, Sigma-Aldrich) were coated with 200 μL anti-rCsGSTo1 or 2 antibodies (1.0 μg/mL) in a carbonate–bicarbonate buffer (100 mM, pH 9.6) overnight at 4 °C. The plates were blocked with phosphate buffered saline containing 0.05% Tween 20 (PBS/T) and 1% bovine serum albumin (BSA) for 1 h at 37 °C, after which, 200 μL rat biliary epithelial extracts (10 μg/mL) were incubated for 2 h at 37 °C. The plates were incubated with 1:1000 diluted *C. sinensis*-infected rat sera (200 μL) for 1 h and subsequently with horse radish peroxidase-conjugated goat anti-rat IgG (1:2000 dilution) for 1 h at 37 °C. Color reaction was developed with Ultra TMB-ELISA substrate (200 μL/well; Thermo Fisher Scientific) in the dark for 10 min and stopped by adding 4 N H_2_SO_4_ (50 μL/well). Absorbance was read at 450 nm. PBS/T was used as a blank control. All results were measured after an appropriate blank correction. Each sample was assayed in triplicate.

### 2.10. Cell Viability

The control HIBECs and rCsGSTos-absorbed HIBECs were incubated with IHBEC-1 medium for 24 h at 37 °C in a 5% CO_2_ incubator in the presence or absence of CHP (50–300 μM). The cells were also exposed to CHP (150 μM) for different time intervals from 30 min to 72 h, and cell viability was spectrophotometrically detected using a D-plus CCK cell viability assay kit (Donginbiotech, Seoul, Korea) at 450 nm (NEO microplate reader, Biotek, Winooski, VT, USA). HIBECs incorporated with heat-inactivated (95 °C for 10 min) rCsGSTos for 1 h (50 µg/mL) were used as controls.

### 2.11. Fluorescence-Activated Cell Sorting (FACS) Analysis

The control HIBECs and HIBECs incorporated with rCsGSTos were stabilized in IHBEC-1 medium for 1 h at 37 °C under 5% CO_2_ atmosphere. Cells were treated with 150 μM CHP and incubated for another 48 h. Cells were harvested with a brief exposure to 0.25% trypsin and washed with PBS (100 mM, pH 7.2). Apoptosis was analyzed by staining with annexin V-APC (Thermo Fisher Scientific) and propidium iodide (PI; Thermo Fisher Scientific) in a FACSCalibur flow cytometry (BD Biosciences, Franklin Lakes, NJ, USA). Annexin V-positive/PI-negative, annexin V-positive/PI-positive and annexin V-negative/PI-positive indicated early apoptosis, late apoptosis and necrosis, respectively. Cytometric data were analyzed using Flowjo software ver10.1 (Ashland, OR, USA).

### 2.12. Western Blot Analysis

Proteins (40 μg/lane) extracted from each conditioned HIBECs were separated by 15% reducing SDS-PAGE. The proteins were electrotransferred to nitrocellulose membrane (Santa Cruz Biotechnology, Dallas, TX, USA) and incubated with the following antibodies (1:1000 dilutions) overnight at 4 °C: p38 MAPK, phosphorylated (p)-p38 MAPK, ERK1/2, p-ERK1/2, JNK, p-JNK, MKK3/6, p-MKK3/6, p-NF-κB p65, Akt, p-Akt, Src, and p-Src (Cell Signaling Technologies, Danvers, MA, USA), and NF-κB p65, PI3K, p-PI3K and β-actin (Abcam). Anti-human GSTo1 (Santa Cruz Biotechnology) was also incubated as above. To observe correlation between differentially expressed mRNA and protein induction profile, antibodies specific to caspase 8 (CASP8; 1:1000 dilution), B-cell lymphoma 2-related protein A1 (BCL2A1; 1:1000 dilution), Fc fragment of IgM receptor (FCMR; 1:500 dilution), colony-stimulating factor 3 (CSF3; 1:500 dilution) and netrin G2 (NTNG2; 1:1000 dilution) were also proved against each conditioned cell lysate as above. Antibodies were purchased from Abcam. Host-specific peroxidase-conjugated anti-IgG antibodies (1:4000 dilution) were incubated for an additional 2 h. Signals were detected by enhanced chemiluminescence (ECL) (GE Healthcare) after 10 min of exposure. The intensity of each band was quantified by ImageJ software (https://imagej.nih.gov/ij/, accessed on 11 November 2020).

### 2.13. Immunohistochemical Staining

*C. sinensis*-infected rat liver specimens were fixed in 4% neutral paraformaldehyde, embedded and cut into 4 μm-thick sections. Deparaffinized sections were permeabilized with 0.5% Triton X-100 and incubated with 10 mM Tris-HCl (pH 8.0) containing proteinase K (20 μg/mL) for 15 min at 37 °C. Sections were blocked by PBS/T containing 3% BSA for 1 h. The slides were incubated overnight with anti-rCsGSTo1, anti-rCsGSTo2 or anti-cytokeratin 19 (CK-19) antibodies (Abcam) diluted to 1:200 and subsequently with FITC-conjugated goat anti-mouse IgG for 2 h (1:500 dilution; Abcam). Nuclear staining was performed using DAPI. Liver sections obtained from uninfected control rat were also subjected to the staining. The images were photographed under a TissueFAXS plus (TissueGnostics, Vienna, Austria).

### 2.14. Statistical Analysis

The experimental results are presented as the mean ± standard deviation (SD). Standard curves of sandwich-ELISA for rCsGSTo proteins were determined by linear regression analysis and correlations were assessed using squared correlation coefficients. The Kolmogorov–Smirnov test was used to obtain GSEA statistics from Quant-RNAseq. Comparison between groups were calculated by Student’s *t*-test using the Excel program (Microsoft, Redmond, WA, USA). Differences were considered to indicate significance at *p < 0.05, ** *p* < 0.01 and *** *p* < 0.001.

## 3. Results

### 3.1. CsGSTo1 and CsGSTo2 Are Accumulated in Host Cholangiocytes In Vivo and In Vitro

**Figure 1:**
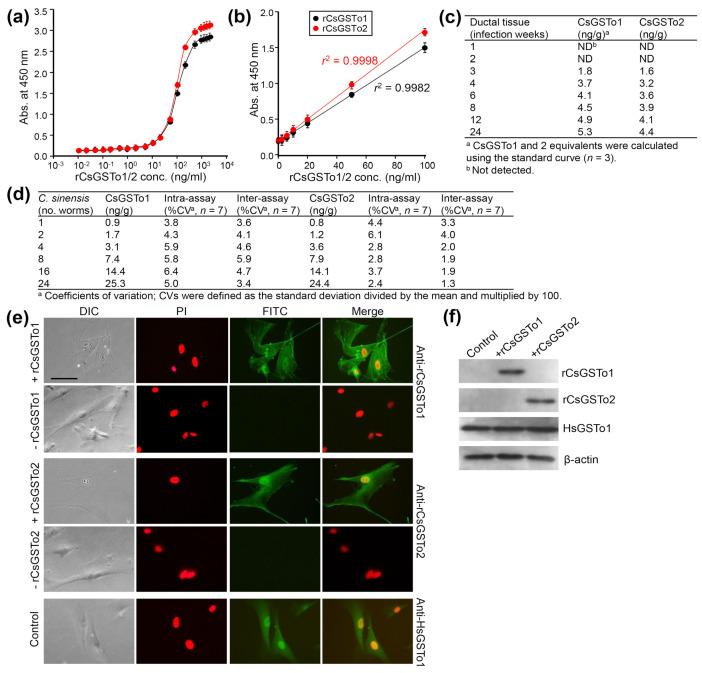
In vivo and in vitro uptake of rCsGSTOs by host cholangiocytes. (**a**) Standard curve of sandwich-ELISA for rCsGSTo proteins. (**b**) Representative linear 8-point calibration curve based on the standard curve. The graph shows the standard deviations of each point and coefficients of variation (r^2^) of triplicate measurements. (**c**) Determination of CsGSTo1 and 2 proteins from the biliary ductal epithelium of rats experimentally infected with *C. sinensis* by the sandwich-ELISA. (**d**) Coefficients of variation for intra- and inter-assay variations. (**e**) Images of HIBECs cultured for 1 h in the presence of rCsGSTo1 or rCsGSTo2 (50 µg/mL), or in the absence of rCsGSTos. Cells were probed with anti-rCsGSTo1, anti-rCsGSTo2 or anti-HsGSTo1 antibodies. Nuclear staining was performed with PI. HsGSTo1 was visualized as an internal control. Bar = 10 µm. (**f**) Accumulation of rCsGSTos in HIBECs shown by Western blotting. HsGSTo1 and β-actin were used for controls.

CsGSTos were released from worms into the host biliary ductal lumen [11]. We asked whether excretory CsGSTos would be absorbed by the host cholangiocytes. We determined the internalization and accumulation of CsGSTos according to the infection duration using biliary ductal epithelium harvested from rats experimentally infected with *C. sinensis*. Standard curves for quantification of rat bile duct epithelial cells were generated in the range of 0.01 to 10,000 ng per ml of rCsGSTo proteins (Figure 1a). The detection limit of the sandwich-ELISA was 0.47 ng per ml for CsGSTo1 and 0.51 ng per ml for CsGSTo2, respectively. The working range achieved linear regression (*r*^2^ = 0.9982 for rCsGSTo1 and *r*^2^ = 0.9998 for rCsGSTo2) for an eight-point calibration curve in the CsGSTo range of 0.1–100 ng per ml (Figure 1b). CsGSTo1 and 2 were detectable from host cholangiocytes at 3 weeks post-infection (1.8 and 1.6 ng/g). These concentrations continually increased until 24 weeks and reached 5.3 and 4.4 ng per g tissue by 24 weeks post-infection (Figure 1c). The coefficients of variation of the intra- and inter-assays were 3.8–6.4% and 3.4–5.9% (CsGSTo1), and 2.4–6.1% and 1.3–4.0% (CsGSTo2), respectively (Figure 1d). We subsequently observed the absorption of rCsGSTos by HIBECs in vitro. When HIBECs were cocultured with different doses of rCsGSTos (10–50 µg/mL), rCsGSTos were incorporated into the cells in a dose-dependent fashion (Figure S1). HIBECs incubated for 1 h in the presence of 50 µg/mL rCsGSTos demonstrated that almost all cholangiocytes (> 98%) exhibited uptake (Figure 1e). Western blot analysis also detected rCsGSTos in the HIBEC cell lysates, together with endogenous human GSTo1 (HsGSTo1) (Figure 1f). This result indicated that excretory CsGSTos were absorbed and accumulated in the host biliary ductal epithelium and further suggested the likelihood of biological effects within these cells.

### 3.2. rCsGSTos Inhibit Oxidative Stress-Induced Apoptosis

**Figure 2:**
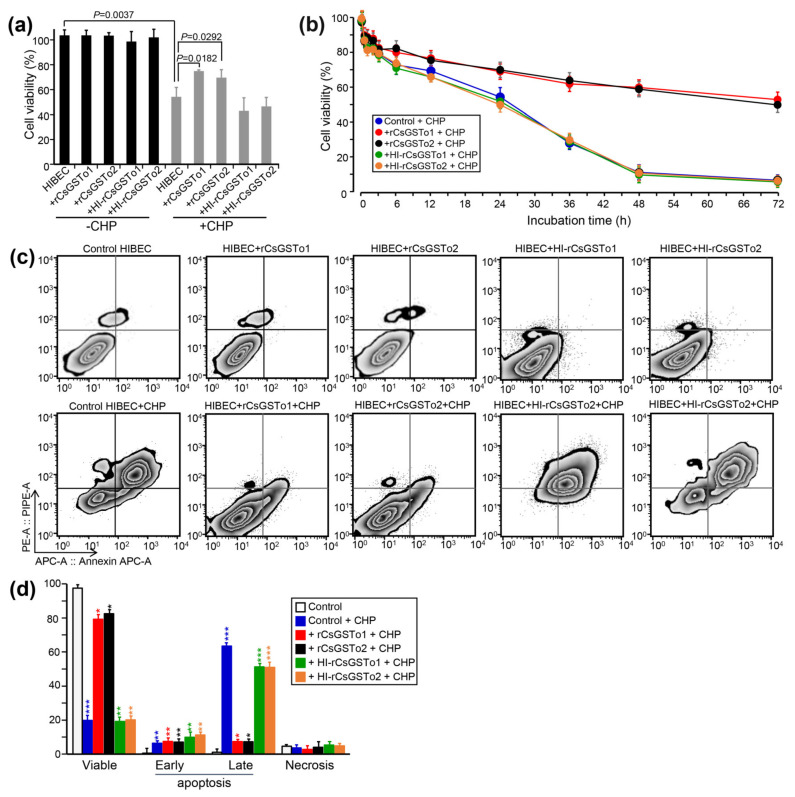
rCsGSTos potentiated cell survival through inhibition of oxidative stress-induced apoptosis. (**a**) Comparison of cell viability between rCsGSTos-incorporated HIBECs and control HIBECs. Control HIBECs and HIBECs introduced with either naïve rCsGSTos or heat-inactivated rCsGSTos were exposed to 150 µM CHP for 24 h at 37 °C in a 5% CO_2_ incubator. Cell viability was spectrophotometrically detected. Mean ± SD (*n* = 6). (**b**) Control and rCsGSTos-incorporated HIBECs were cultured for different time intervals in the presence of 150 µM CHP. HIBECs incorporated with heat-inactivated rCsGSTos were also assayed. Mean ± SD (*n* = 3) * *p* < 0.05, ** *p* < 0.01, *** *p* < 0.001. (**c**) rCsGSTos significantly rescued human cholangiocyte from oxidative stress-induced apoptosis. HIBECs introduced with either naïve rCsGSTos or heat-inactivated rCsGSTos were incubated for 24 h in the presence of 150 µM CHP. Apoptosis rates were analyzed by FACS. The cells were stained with annexin V (Annexin APC-A) and propidium iodide (PE-A). (**d**) Statistical analysis of early and late apoptosis rates, and necrosis in control and rCsGSTos-incorporated HIBECs. Mean ± SD (*n* = 3). * *p* < 0.05, ** *p* < 0.01, *** *p* < 0.001.

CsGSTos were reported to exert an important role in defending the parasite from exogenous stresses [13]. We examined whether CsGSTos accumulated in the host cholangiocytes would protect cells from oxidative stress-induced cytotoxicity. When control cells and HIBECs incorporated with naïve rCsGSTos or heat-inactivated rCsGSTos were incubated for 1 h, proportions of viable cells were similarly observed (103.5–103.8%, *p* > 0.05). We exposed cells to 150 µM CHP for 24 h to create an oxidative stress and observed a significant loss of cell viability compared to non-stressed control (54.2% vs. 111.5%, *p* = 0.0037). Conversely, the incorporation of naïve rCsGSTo1 or 2 rescued cell viability to levels that were 71.8% (*p* = 0.0182) or 69.5% (*p* = 0.0292) of baseline by preventing CHP-induced cytotoxicity, whereas heat-inactivated rCsGSTos did not show any effect on cell survival (Figure 2a,b). A time-course analysis demonstrated that this prophylactic activity became more obvious over longer durations. At 72 h of incubation, the cell viability of control HIBECs was 5.9%, while those rCsGSTos-incorporated cells maintained 54.6% and 55.7% viability (*p* < 0.01), respectively. During the incorporation of rCsGSTos into HIBECs, we incubated cells in serum-free media for 3 h, but cell viability was not significantly affected (*p* > 0.05) (Figure 2b). We exposed cells to 150 µM CHP for 48 h and determined the apoptosis rate. CHP treatment substantially increased the apoptosis rate in control cells compared to non-stressed cells (early apoptosis; 6.34 ± 1.28% vs. 0.50 ± 0.08%, *p* = 0.0081; late apoptosis; 63.37 ± 1.14% vs. 1.04 ± 4.27%, *p* < 0.001). HIBECs incorporated with heat-inactivated rCsGSTo showed apoptosis rates similar to those of control cells. However, the incorporation of naïve rCsGSTos remarkably salvaged the cells by inhibiting early (rCsGSTo1; 5.41 ± 0.68%; rCsGSTo2; 4.03 ± 0.59%, *p* = 0.0284) and late apoptosis (rCsGSTo1; 7.33 ± 0.92%; rCsGSTo2; 7.21 ± 1.02%, *p* < 0.001) (Figure 2c,d). These results demonstrated that rCsGSTos play pertinent roles in scavenging ROS (reactive oxygen species) generated by CHP, and thus contribute to an alleviation of cholangiocyte cytotoxicity.

### 3.3. Functional Characterization of the Altered HIBEC Intracellular Environment in Response to rCsGSTos

**Figure 3:**
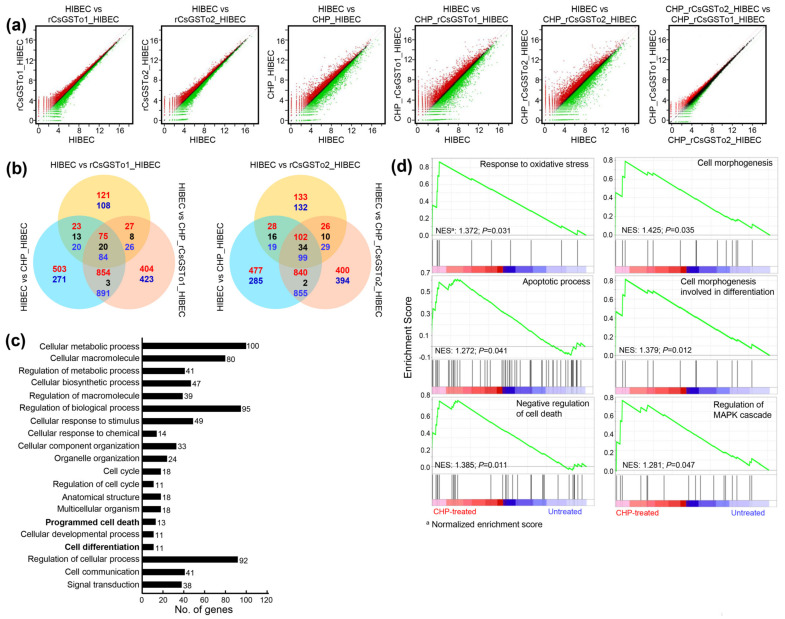
rCsGSTos induced transcriptomic changes in HIBECs upon oxidative stimulus. (**a**) Scatter plots of microarray defined genes showing expressional changes. Control and rCsGSTos-incorporated HIBECs were incubated in the presence or absence of CHP for 24 h and analyzed by Quant-RNAseq. The raw reads of normal control were > 22.2 M and those of cells incorporated with rCsGSTos without stress were > 24.8 M (CsGSTo1) and > 20.7 M (CsGSTo2). The raw reads of control cells and rCsGSTo-incorporated cells exposed to stress were > 13.7 M, > 18.4 M (CsGSTo1) and > 18.0 M (CsGSTo2), respectively. The plot is on a log2 transformed scale. (**b**) Venn diagram shows the number of differentially expressed genes. Overlap of each set of genes is shown. Red, blue and black letters indicate the number of up-, down- and contra-regulated genes, respectively. (**c**) Quant-RNAseq array defined distribution of genes related to cellular process in biological process at tertiary hierarchy are presented by Blast2GO. (**d**) Among 412 genes showing significant transcriptional alteration by GSEA (*p* < 0.05), genes functionally engaged in response to oxidative stress, apoptotic process, negative regulation of cell death, cell morphogenesis, cell morphogenesis involved in differentiation and regulation of MAPK cascade were analyzed.

We sought to define the molecular events underlying this cellular protection of rCsGSTos-incorporated HIBECs against oxidative stressful conditions through Quant-RNAseq array in comparison with the control. We identified 25,737 transcripts that showed differential expression patterns (Supplementary Data Sheet 1). Spreading spots of scatter plots of CHP-treated rCsGSTos-absorbed HIBECs showed the larger expression differences, but distributional changes of the transcriptome profiles of HIBECs between rCsGSTo1 and rCsGSTo2 incorporation was not significantly different from each other (Figure 3a). When transcriptional changes of CHP-treated rCsGSTo1-incorporated HIBECs were compared to those of rCsGSTo2-incorporated cells, 263 genes exhibited altered transcription (263/25,737 genes, 1.02%); an increase in 111 and decrease in 152 genes. rCsGSTo1 or 2 largely modified transcription levels of 229 or 265 genes compared to the control cells. This singularity was more profoundly apparent when these cells were exposed to oxidative stress. A total of 2575 and 2491 transcripts displayed differential expression patterns (Figure 3b).

To delineate the functional relevance of rCsGSTos within host cholangiocytes, we extracted 412 genes that displayed significant transcriptional alterations upon oxidative injury (*p* < 0.05); 147 genes were upregulated and 265 genes downregulated (Figure S2a and Supplementary Data Sheet 1). The assignment of gene ontology terms by Blast2GO demonstrated that CsGSTos affected a broad spectrum of biological process, molecular function and cellular component. In the biological process, genes associated with the cellular process, metabolic process, biological regulation and response to stimulus constituted major fractions. The main components composing molecular function were binding and catalytic activity. The cellular component was largely allocated to cellular anatomical entity, intracellular and protein-containing complex (Figure S2b).

We dissected ontology terms of RNAseq data assigned to the cellular process within the biological process at tertiary hierarchy (Figure 3c) and identified the KEGG pathways of 412 mRNAs that revealed transcriptional alterations (Table 1). A total of 173 GO classes showing significant alteration were detected by GSEA (nominal *p*-value < 0.05) (Supplementary Data Sheet 2). Since the incorporation of rCsGSTos significantly protected HIBECs from CHP-induced apoptosis (Figure 2c,d), we selected genes associated with response to oxidative stress, apoptotic process, the negative regulation of cell death and the regulation of MAPK cascade. We also included genes related to cell morphogenesis involved in differentiation, because the loss of programmed cell death might disturb structural and biochemical plasticity of cholangiocytes [22]. Most of these genes enriched at the core of each GO classification (Supplementary Data Sheet 3) were similarly distributed in GSEA. The 17 genes, which were classified as response to oxidative stress stimulus by Blast2GO, were grouped as the regulation of the MAPK cascade. The 13 genes associated with programmed cell death were categorized into the apoptosis process and the negative regulation of cell death by GSEA; all of these genes were involved in cell survival (Table 1). Out of 11 genes related to cell differentiation by Blast2GO, two genes were classified into cell morphogenesis involved in differentiation (Figure 3d), while nine genes could not be properly determined by GSEA. These collective results implied that rCsGSTos might induce transcriptional and translational changes in genes involved in apoptosis and differentiation via the modulation of MAPK signaling, and by these routes participate in the regulation of intracellular protective functions to cope with oxidative insult.

### 3.4. rCsGSTos Augment Cholangiocyte Survival through Activation of Src-PI3K/Akt-NF-κB p65 Signal Pathway Followed by Expressional Regulation of Genes Engaged in Apoptotic Process

**Figure 4:**
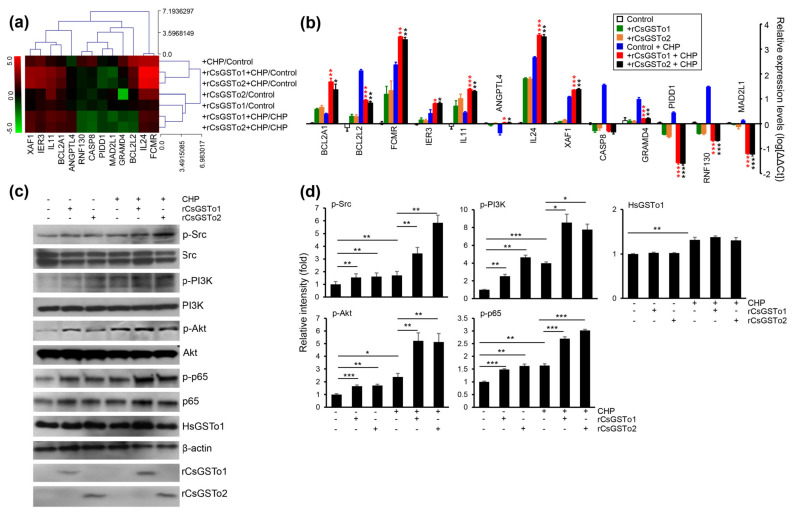
rCsGSTos increased active, phosphorylated forms of protein kinases involved in cell survival followed by controlling the transcriptional levels of apoptotic genes in response to oxidative injury. (**a**) A clustergram of genes involved in programmed cell death. Red and green in the hit map indicated maximal and minimal gene expression. (**b**) Transcription levels of genes shown in panel a were determined by qRT-PCR with gene-specific primers (Table S1). β-actin gene was used as an internal control for normalization. * *p* < 0.05, ** *p* < 0.01, *** *p* < 0.001. (**c**) Western blot analysis of phosphorylated forms of Src, PI3K, Akt, and NF-κB p65. Detection of β-actin and rCsGSTos is also shown. (**d**) Relative fold intensity of kinases activated by phosphorylation. The intensity of Western blot bands was quantified by ImageJ analysis (https://imagej.nih.gov/ij/, accessed on 31 March 2020). * *p* < 0.05, ** *p* < 0.01, *** *p* < 0.001.

We identified 13 genes involved in programmed cell death which were modified as a part of the oxidative stress response (apoptosis process and negative regulation of cell death by GSEA); seven antiapoptotic genes, i.e., angiopoietin-like 4 (ANGPTL4), B-cell lymphoma 2-related protein A1 (BCL2A1), BCL2-like 2 (BCL2L2), Fc fragment of IgM receptor (FCMR), immediate early response 3 (IER3), ring finger protein 130 (RNF130) and interleukin 11 (IL11). The six apoptotic genes included caspase 8 (CASP8), GRAM domain containing 4 (GRAMD4), interleukin 24 (IL24), MAD1 mitotic arrest deficient-like 1 (MAD2L1), p53-induced death domain protein 1 (PIDD1) and X-linked inhibitor of apoptosis protein associated factor 1 (XAF1) (Figure 4a). qRT-PCR analysis demonstrated that transcription levels of four antiapoptotic genes (IER3, IL11, FCMR and BCL2A1) and two apoptotic genes (IL24 and XAF1) were increased in rCsGSTos-incorporated HIBECs. The transcription of antiapoptotic ANGPTL4 was decreased in the control HIBECs exposed to CHP, but its expression was recovered by rCsGSTos. Two antiapoptotic genes, BCL2L2 and RNF130, and three apoptotic genes, GRAMD4, CASP8 and PIDD1, were upregulated in the control HIBECs treated with CHP, but downregulated by rCsGSTos. rCsGSTos-introduced HIBECs showed the decrease in the transcription of the apoptotic gene, MAD2L1. Transcripts of BCL2A1 and BCL2L2 were inversely regulated by rCsGSTos; with increases to BCL2A1 and decreases to BCL2L2 (Figure 4b). When we examined the changes of CASP8, BCL2A1 and FCMR gene expression at the protein level, immunoblotting outcomes were correlated with those of mRNAs (Figure S3a,b).

The assessment of KEGG pathways and GSEA demonstrated that genes involved in anti-apoptosis and cell survival were substantially enriched (Table 1 and Figure 3d). We examined kinase activity and observed that active, phosphorylated forms of Src, PI3K/Akt and NF-κB p65 were increased by the incorporation of rCsGSTos (Figure 4c,d). These results strongly suggest that rCsGSTos assimilated into the host cholangiocytes augment cell survival through the phosphorylation activation of Src at an upstream position followed by the progressive activation of downstream PI3K/Akt and NF-κB p65 signaling.

### 3.5. rCsGSTos Induce Phosphorylation of MKK3/6 and p38 MAPK, thus Regulate the Expression of Genes Involved in Cell Differentiation and Are Located in the C. sinensis Infected Biliary Ductal Epithelium Where Cytokeratin 19 Is Expressed

**Figure 5:**
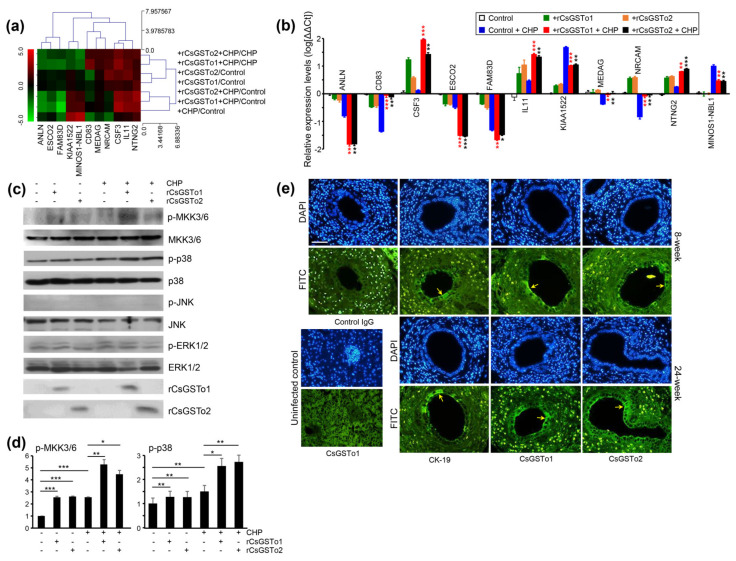
rCsGSTos regulated protein kinase activity as well as controlled transcriptional levels of genes associated with cell differentiation and are distributed in *C. sinensis*-infected rat biliary epithelium together with CK-19. (**a**) Expressional changes of gene cluster. RNAs isolated from each conditioned cell were subjected to Quant-RNAseq array. (**b**) qRT-PCR analysis of gene cluster shown in panel a. β-actin gene was used as the internal control for normalization. * *p* < 0.05, ** *p* < 0.01, *** *p* < 0.001. (**c**) Western blot analysis of phosphorylation of MKK3/6, p38, JNK and ERK1/2. (**d**) Increase in phosphorylated forms of protein kinases analyzed by relative fold intensity. The intensity of respective bands was quantified by ImageJ analysis (https://imagej.nih.gov/ij/, accessed on 11 November 2020). * *p* < 0.05, ** *p* < 0.01, *** *p* < 0.001. (**e**) Distribution of CsGSTos and CK-19 in *C. sinensis*-infected rat biliary ductal epithelium. Sections were probed with anti-CK-19, anti-rCsGSTo1 or 2 antibodies. Along with infection duration, CsGSTo accumulates were increased and the distribution pattern was consistent with CK-19 (arrows). Nuclear staining was carried out with DAPI. Anti-CK-19 and control IgG were used as cell differentiation marker and negative control, respectively. Liver specimens from uninfected rat was used to examine the absence of CsGSTo proteins. Immunostaining using anti-rCsGSTo1 is shown. Eight- and 24-week each denotes post-infection weeks. Bar = 10 µm.

Transcriptomic changes associated with the cell differentiation (cell morphogenesis involved in differentiation by GSEA) of rCsGSTos-incorporated HIBECs revealed 11 genes that were significantly modified in response to oxidative stress (Figure 5a). The expression levels of colony-stimulating factor 3 (CSF3), IL11 and netrin G2 (NTNG2) were increased. In contrast, the expression of anillin (ANLN), establishment of sister chromatid cohesion N-acetyltransferase 2 (ESCO2), uncharacterized protein KIAA1522 (KIAA1522), neuroblastoma suppressor of tumorigenicity 1 (MINOS1-NBL1) and family with sequence similarity 83 member D (FAM83D) were decreased. The transcription levels of a cluster of differentiation 83 (CD83), mesenteric estrogen-dependent adipogenesis (MEDAG) and neuronal cell adhesion molecule (NRCAM), which were reduced by CHP treatment, were recovered by supplementing rCsGSTos (Figure 5b). The expression of CSF3 and NTNG2 proteins was parallelly regulated with those of mRNAs (Figure S3a,b).

When we examined the activation of signal molecules, phosphorylated forms of MKK3/6 and p38 MAPK were increased, while those of JNK and ERK1/2 remained unchanged (Figure 5c,d). To assess whether cell differentiation is associated with cholangiocytes, we localized CK-19 and CsGSTo molecules in the *C. sinensis*-infected rat liver sections. CK-19 was distributed in the biliary ductal epithelium where CsGSTos were accumulated. Along with the progression of infection, CsGSTo increasingly accumulated with the distributional pattern, consistent with CK-19 (Figure 5e). These results demonstrated that CsGSTo accumulation increased as inflammation-associated oxidative stress persisted chronically and further suggested that CsGSTos assimilated by host cholangiocytes induced the phosphorylation of MKK3/6 and p38 MAPK.

## 4. Discussion

The perturbation of programmed cell death [23,24,25] and progressive development of precancerous lesions, such as metaplasia, dysplasia and hyperplasia of cholangiocytes [2,26] are the characteristic histopathological features of chronic clonorchiasis. To understand the mechanisms underlying these phenomena, we investigated the biological consequences of the incorporation and accumulation of CsGSTos in the host biliary ductal epithelium. CsGSTos assimilated within host cholangiocytes appear to augment anti-apoptosis through the increase in the active, phosphorylated forms of Src, PI3K/Akt and NF-κB p65 and the subsequent modulation of several genes involved in programmed cell death. These enzymes also induce cholangiocyte differentiation through control of the expression of responsible genes via the activation of MKK3/6 and p38 MAPK.

We previously reported that *E. coli* cells transfected with CsGSTo expression plasmids were resistant to oxidative killing [13]. A mutant *Caenorhabditis elegans* that had introduced double-stranded GSTo RNA (GST-3) of a parasitic nematode *Onchocerca volvulus* also showed oxidative stress resistance [27]. These results indicate that the ectopically expressed enzymes were able to protect host cells from oxidative injuries. During the course of *C. sinensis* infection, CsGSTos are secreted from the worms in the host [11,13], but the cellular uptake and biological significance of these enzymes within the host cholangiocytes have not been explored. We integrated rCsGSTos into HIBECs derived from normal human biliary ductal epithelium [21] and observed intracellular events as a consequence. Host cholangiocytes appear to absorb CsGSTos from the biliary lumen by osmotic gradient differences, just as they absorbed several resources (i.e., amino acids, glucose, bile acids and water) from bile [22]. Our choice of HIBECs assumed that pathophysiological changes occurring in normal cholangiocytes would represent in vivo responses better than in the modified cell lines, such as HuCCT1 or H69 [18,19,28].

The knock-down of endogenous HsGSTo1 in HIBECs employing short-hairpin RNA significantly reduced cell viability, while the introduction of rCsGSTos with these cells greatly restored, although not completely, cell survival (Figure S4a–d). This result demonstrated that rCsGSTo protected the cells by compensating for HsGSTo1 function. However, CsGSTos accumulated in the cholangiocytes carrying HsGSTo1 in the clinical course of human clonorchiasis. We therefore conducted the following experiments using HIBECs without knock-down HsGSTo1 to mimic the natural host microenvironment. rCsGSTos partially but significantly salvaged host cholangiocytes from oxidative insult by inhibiting apoptosis.

The incorporation of rCsGSTos within HIBECs induced transcriptional changes in tens of thousands of genes and this response became more evident when cells underwent oxidative stressful condition. We characterized genes associated with programed cell death and cell differentiation while analyzing GO, KEGG pathways and GSEA of RNA microarray. In response to oxidative stress, the transcription of 13 genes associated with programmed cell death was significantly altered. qRT-PCR analysis demonstrated that the changes favored anti-apoptosis pathways; increases in antiapoptotic genes and reductions in apoptotic genes. The expression of some genes was inversely controlled, but this modification was also mostly directed towards suppressing apoptosis. BCL2A1 and IER3 transcripts were highly expressed in CHP-treated HIBECs supplemented with rCsGSTos. BCL2A1 targets to NF-κB, thus enhancing anti-apoptosis and cell survival [29]. IER3 leads to cell survival by reducing intracellular ROS levels [30]. Tumor necrosis factor-α-induced apoptosis of NF-κB p65 knock-out was reversed in the presence of IER3 [31]. The transcription levels of apoptosis-related genes, which included CASP8 and PIDD1 [32], GRAMD4 [33] and XAF1 [34] were upregulated in control cells upon treatment with CHP, while their expressions were downregulated by rCsGSTos. Although we did not analyze the differential expression profile of all these genes at the protein level, the proteins we examined were regulated in a manner similar to those of mRNAs. We surmise that the mRNA quantification of differentially expressed genes may reflect expressional changes of the proteins [35]. The analysis of protein kinases involved in cell survival revealed increases in active, phosphorylated forms of Src, PI3K, Akt and NF-κB p65 molecules. These data collectively indicated that rCsGSTos absorbed in the host cholangiocytes inhibit apoptosis and enhance cellular survival through the modulation of responsible genes following the activation of protein kinases.

An earlier study with *O. viverrini* thioredoxin-1 (Ov-Trx-1) reported that the incorporation of Ov-Trx-1 in H69 cells suppressed the expression of apoptosis-related genes followed by the blockade of the MAPK/apoptosis signal-regulating kinase-1 signal pathway. Ov-Trx-1 might directly scavenge ROS through the increases in the cytosolic thioredoxin pool [28,36]. The inhibition of sigma-class GST-mediated prostaglandin synthesis by a stilbene polyphenol, resveratrol [37], attenuated cholangiocyte tumorigenesis in hamsters infected with *O. felineus* through the suppression of cyclooxygenase and hydroperoxidase functions [6]. Mammalian GSTo1 inhibits JNK via the regulation of glutathionylation of the target proteins [16]. However, the GSTo-mediated activation of Src, PI3K/Akt and NF-κB p65 signaling pathways and the subsequent regulation of transcription and translation of genes have not been addressed. GSTo seems to utilize different molecular machinery to induce the antiapoptotic process, resulting in a greater cell survival.

We analyzed the genes associated with cell differentiation and found 11 genes showed altered transcription. CSF3, IL11 and NTNG2, whose functions are principally involved in anti-apoptosis and differentiation [38,39], were increased. The downregulated expression of NRCAM, which induces cell–cell adhesion in differentiating cells [40], was recovered by supplementing rCsGSTos. The induction profile of these proteins was reflected in the changes of mRNA expression. Western blot analysis detected active, phosphorylated forms of MKK3/6 and p38 MAPK. The MKK3/6-p38 signal pathway executes multifunctional impacts in apoptosis, differentiation, migration and adhesion, not only in diverse cellular homeostatic processes, but also in pathological conditions [41,42]. p38 MAPK promotes apoptosis in conjunction with tumor suppressors and caspases. However, we could not observe increases in the transcription level of tumor suppressing p53 gene (TP53) and caspase signaling pathways in HIBECs incorporated with rCsGSTos. Furthermore, phosphorylated forms of JNK, the activation of which is related to apoptosis [41], and ERK1/2, which enhances apoptosis upon oxidative injuries [43], were not detected. Given that ROS directly activates ERK1/2 [44], CsGSTos might suppress ERK1/2 activation through the inhibition of ROS production. These results demonstrated that the activation of MKK3/6 and p38 MAPK might be associated with cell differentiation, but not with apoptotic process [45,46,47]. Indeed, CK-19, a well-known biliary marker for cell differentiation [48], was distributed in the biliary ductal epithelium of *C. sinensis*-infected rats, where excretory CsGSTos were accumulated. This event was more evident as infection progressed.

Transforming growth factor-β, Notch and Wnt/β-catenin signaling in biliary ductal differentiation in early developmental stages have been well described [49], but we could not obtain any evidence for the activation of such molecules. This result further suggested that the induction of MKK3/6 and subsequent activation of p38 MAPK followed by ductal differentiation might be differentially regulated to meet the pathobiological adaptations occurred in clonorchiasis. Our data suggest novel pathways through which CsGSTos potentiate both anti-apoptosis and the differentiation of cholangiocytes.

In this study, we observed reductions in ANLN, ESCO2 and FAM83 transcripts upon treatment with CHP, while these genes are known to induce cell differentiation [50,51,52]. It is not clear at present how to reconcile these apparently conflicting results, which await further elucidation. Potentially, these genes may be bystanders in the differentiation of the biliary ductal epithelium. It is not known whether CsGSTos directly induce the phosphorylation of Src and MKK3/6 molecules or whether other mediators are involved in this process. The metabolites produced by lipid peroxidation might influence the activity of transcription factors and protein kinases associated with stress responses, proliferation, differentiation or apoptosis [53]. These intriguing issues should form the basis for future studies.

Anti-apoptosis and cell differentiation are characteristic features of malignant transformation and are not occurred independently, but are highly coordinated processes in which diverse transcriptional and signaling machineries stimulated by certain factor(s) are orchestrated. We demonstrated that excretory CsGSTos are internalized into the host biliary ductal epithelium and activate protein kinases involved in apoptosis and cell differentiation followed by the regulation of target genes in response to oxidative stress. Our results have brought new insights into the cross-talk between CsGSTos and host cholangiocytes during host–parasite interplay.

## 5. Conclusions

**Figure 6:**
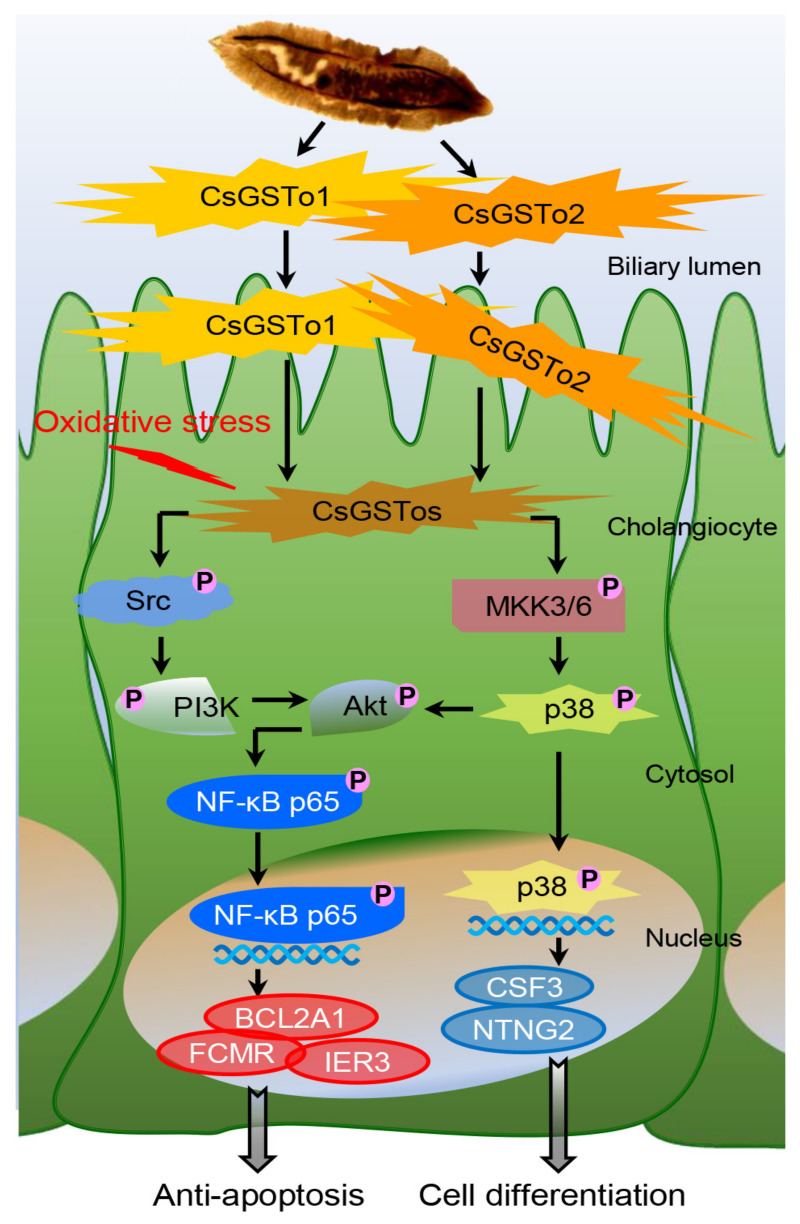
CsGSTo-mediated novel pathways for survival and differentiation of cholangiocyte in response to oxidative stress. CsGSTos excreted from *C. sinensis* were released into the biliary ductal lumen and then absorbed into the host cholangiocytes. When cholangiocytes are triggered by stress, CsGSTos activated upstream Src and downstream PI3K/Akt and NF-κB p65 molecules. Phosphorylated NF-κB p65 translocated into the nucleus and modulated expression of genes involved in apoptotic process. CsGSTos also increased phosphorylation of MKK3/6 followed by p38 MAPK. Subsequent nuclear translocation of activated p38 modified expression of target mRNAs and proteins associated with cell differentiation.

On the basis of our collective findings, we propose novel mechanisms for CsGSTos-mediated survival and the differentiation of cholangiocytes in response to oxidative stress. CsGSTos excreted into the biliary ductal lumen are internalized to the host cholangiocytes. When cholangiocytes are triggered by stress, CsGSTos activate Src, PI3K/Akt and NF-κB p65 signal molecules. Phosphorylated NF-κB p65 migrates to the nucleus and modulates the expression of genes involved in apoptosis. CsGSTos also induce the phosphorylation of MKK3/6 followed by p38 MAPK. The subsequent nuclear translocation of activated p38 controls the expression of target genes associated with cell differentiation (Figure 6). The targeting of CsGST proteins may prove beneficial to prevent clonorchiasis-related CCA tumorigenesis.

## Figures and Tables

**Table 1:** Analysis of 412 genes involved in signaling pathways that showed altered expression by rCsGSTos under oxidative stress.

KEGG ID	Signaling Pathways ^a^	No. of Gene	Related Genes
hsa04621	NOD-like receptor	10	NEK7, CXCL1, CXCL3, IRF7, OAS1, OAS2, OAS3, CCL5, STAT1, **CASP8**^b^
hsa04064	NF-kappa B	9	GADD45G, EDARADD, CXCL1, CXCL3, ICAM1, PIDD1, PTGS2, **BCL2A1**, TNFSF11
hsa04010	MAPK	8	GADD45G, DUSP8, EPHA2, MECOM, HSPA1L, HSPA6, MAPK7, MAPK8IP1
hsa04657	IL-17	7	**CSF3**^c^, CXCL1, CXCL3, IL17D, MAPK7, PTGS2, **CASP8**
hsa04115	p53	6	GADD45G, CD82, **PIDD1**, **CASP8**, CCNB2, CDK1
hsa04668	TNF	6	CXCL1, CXCL3, ICAM1, PTGS2, CCL5, **CASP8**
hsa04630	JAK/STAT	5	**IL24**, **CSF3**, **IL11**, IL17D, STAT1
hsa04071	Sphingolipid	5	S1PR1, S1PR3, CERS2, ABCC1, BDKRB2
hsa04020	Calcium	4	AGTR1, EDNRB, PHKG2, BDKRB2
hsa04620	Toll-like receptor	4	IRF7, CCL5, STAT1, **CASP8**
hsa04922	Glucagon	4	SIK1, CRTC2, PHKG2, PPP4C
hsa04915	Estrogen	4	HBEGF, HSPA1L, HSPA6, KRT15
hsa04062	Chemokine	4	CXCL1, CXCL3, CCL5, STAT1
hsa04625	C-type lectin receptor	4	IL17D, PTGS2, STAT1, **CASP8**
hsa04068	FoxO	4	PLK4, GADD45G, S1PR1, CCNB2
hsa04622	RIG-I-like receptor	3	IRF7, **CASP8**, ISG15
hsa04151	PI3K/Akt	3	**CSF3**, EPHA2, CRTC2
hsa03320	PPAR	3	**ANGPTL4**, ACSL5, PPARG
hsa04022	cGMP/PKG	3	AGTR1, EDNRB, BDKRB2
hsa04921	Oxytocin	3	KCNJ2, MAPK7, PTGS2

^a^ Signaling pathways were extracted from KEGG mapper (https://www.genome.jp/kegg/mapper.html, accessed on 1 April 2020). ^b^ Genes showing downregulated expression are marked by red-bolded letters. ^c^ Genes showing upregulated expression are marked by bolded letters.

## Data Availability

The data presented in this study are available within the article and its Supplementary Materials. Other data that support the findings of this study are available upon request from the corresponding author.

## Supplementary Materials

The following are available online at https://www.mdpi.com/article/10.3390/antiox10071017/s1.
